# Pathogenic Biohacking: Induction, Modulation and Subversion of Host Transcriptional Responses by *Listeria monocytogenes*

**DOI:** 10.3390/toxins12050294

**Published:** 2020-05-05

**Authors:** Matthew J. G. Eldridge, Pascale Cossart, Mélanie A. Hamon

**Affiliations:** 1 Chromatine et Infection G5, Institut Pasteur, 75015 Paris, France; matthew.eldridge@pasteur.fr; 2 Unité des Interactions Bactéries-Cellules, Institut Pasteur, 75015 Paris, France; pascale.cossart@pasteur.fr

**Keywords:** gene expression, transcription factor, epigenetics, *Listeria*, virulence factor

## Abstract

During infection, the foodborne bacterial pathogen *Listeria monocytogenes* dynamically influences the gene expression profile of host cells. Infection-induced transcriptional changes are a typical feature of the host-response to bacteria and contribute to the activation of protective genes such as inflammatory cytokines. However, by using specialized virulence factors, bacterial pathogens can target signaling pathways, transcription factors, and epigenetic mechanisms to alter host gene expression, thereby reprogramming the response to infection. Therefore, the transcriptional profile that is established in the host is delicately balanced between antibacterial responses and pathogenesis, where any change in host gene expression might significantly influence the outcome of infection. In this review, we discuss the known transcriptional and epigenetic processes that are engaged during *Listeria monocytogenes* infection, the virulence factors that can remodel them, and the impact these processes have on the outcome of infection.

## 1. Introduction

Regulation of gene expression at the transcriptional level is fundamental in enabling a cell to enact transient or permanent phenotypic changes. Through the actions of intricate networks of receptors and signaling cascades, cells integrate external signals into a transcriptional response in order to adapt to environmental stimuli. Signal transduction pathways relay information to distinct transcriptional and epigenetic regulators, that ensure the necessary alterations in gene expression. Given its importance, transcription is tightly regulated at multiple levels by transcription factors and chromatin modifiers. Chromatin is a complex of DNA and protein and plays an important role in organizing the genetic material of the cell. The primary structural component of chromatin is the nucleosome, which consists of DNA wrapped around a protein octamer comprising of core histones H2A, H2B, H3, and H4 [1]. Chromatin modifiers such as histone-modifying enzymes, and chromatin-remodeling enzymes, regulate the accessibility of DNA to transcription factors and the transcriptional machinery. Therefore, chromatin modifications are essential for signal transduction integration into a transcriptional response, as they control whether a gene is held in an active or silent state [1,2].

Post-translational modification (PTM) of histones can regulate the transcriptional permissiveness of chromatin by inducing direct structural alterations, or recruiting additional chromatin-regulating factors [3,4]. Among the most well-defined modifications are acetylation, phosphorylation and methylation, which occur on histone N-terminal tails and select residues in the globular domain [5]. Histone acetylation and phosphorylation promote transcription by disrupting the electrostatic interactions between histones and DNA, thereby relaxing chromatin structure and making DNA more accessible to transcription factors. On the contrary, the absence or removal of such modifications can have the opposing effect, causing chromatin compaction and transcriptional repression [3,6]. In addition to a structural role, histone modifications also act as binding platforms to facilitate or block recruitment of other chromatin-regulating proteins [7,8,9,10], including transcription factors [11,12,13], chromatin remodeling enzymes [13], histone/DNA-modifying enzymes [13,14], or protein complex adaptors [13,15]. Histone modifications are often dynamic and, in some cases, only occur transiently to promote the transcription of particular genes. However, certain repressive histone marks recruit proteins such as heterochromatin protein 1 (HP1), which holds chromatin in its condensed and silent configuration, and has more stable, long lasting effects [7,16,17]. Gene regulators, such as transcription factors and epigenetic processes, work concurrently to ensure appropriate and robust transcriptional regulation in response to environmental stimuli and stress, such as bacteria.

The bacterium *Listeria monocytogenes* (*Lm*) is a well-established model organism in the study of host–pathogen interactions and has been used extensively to examine host transcriptional responses to infection [18,19,20,21,22,23,24]. *Lm* is a foodborne, facultative intracellular pathogen and causative agent of listeriosis, which typically manifests as febrile, self-limiting gastroenteritis [24]. However, *Lm* can also cross cellular barriers to cause systemic disease, which can be fatal in immunocompromised individuals, and cause dangerous prenatal infections in pregnant women. *Lm* is transmitted orally, usually via contaminated food, and enters the gastrointestinal tract where it invades and replicates within epithelial cells [25]. During systemic infection, *Lm* breaches the intestinal barrier, enters the bloodstream and disseminates to secondary infection sites such as the liver and spleen. Because of its ability to invade non-phagocytic cells, *Lm* can also cross endothelial barriers, such as the blood–brain barrier or placenta, causing sepsis, meningitis, or miscarriage.

During infection, *Lm* comes into contact with many different tissues and cell types, and therefore causes widespread changes to the gene expression profile of its host, both at a cellular [18,19,20,21], and whole organism level [22,23]. *Lm* is detected by multiple families of pattern recognition receptor (PRR), including the nucleotide-binding domain and leucine-rich repeat (NLR) proteins, and toll-like receptors (TLRs), which respectively localize to the cytoplasm and cellular membranes. These receptors play a crucial role in generating the transcriptional response to *Lm* by signaling to transcription factors such as nuclear factor-κB (NF-κB) [26], which regulates the expression of hundreds of genes important for inflammatory and immune responses [27,28,29]. Later stages of infection are also characterized by the activation of additional signaling cascades leading to global changes in transcriptional regulation of the host [18,19,20,21,22,26]. In addition to the transcriptional response that follows the detection of bacteria, *Lm* also actively reprograms the host response with virulence factors thereby promoting its own ability to survive and cause disease [30,31,32,33,34]. In this review, we will focus on the transcriptional and epigenetic changes that arise upon sensing *Lm* during infection, the mechanisms used by *Lm* to influence these events, and how these illustrate the complex signaling crosstalk that takes place during infection.

## 2. Host-Driven Antimicrobial Inflammatory Responses

Transcriptional responses to *Lm* infection include the induction of two broad categories of gene expression profiles: one dominated by NF-κB and Mitogen-activated protein kinase (MAPK)-regulated inflammatory genes; and another composed of interferon-stimulated genes (ISGs) [24,29,35].

### 2.1. NF-κB and MAP Kinase-Regulated Transcriptional Profile

**Figure 1:**
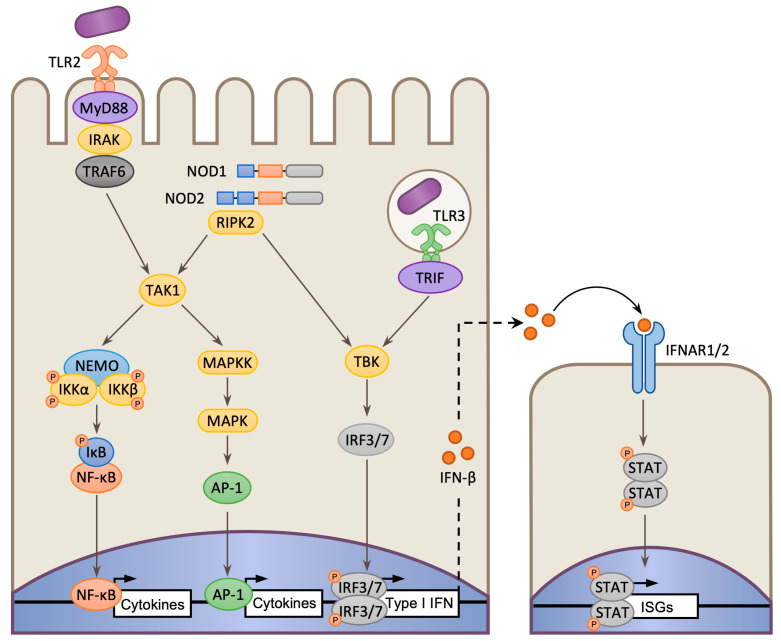
Toll-like receptors (TLR) and nucleotide-binding domain and leucine-rich repeat (NLR) receptor signaling in response to *L. monocytogenes* (*Lm*). TLR2 recognizes lipoproteins (LP) or lipoteichoic acids (LTA) and transmits via a signal transduction pathway including Myeloid differentiation primary response 88 (MyD88), IL-1R-associated kinase (IRAK) proteins and TNF receptor-associated factor 6 (TRAF6). NOD1 and NOD2 recognize diaminopimelic acid (DAP) and muramyl dipeptide (MDP) respectively, resulting in RIPK2 recruitment and activation. These converge on TGFβ-activated kinase 1 (TAK1) which triggers two diverging pathways: activation of the IκB kinase (IKK) complex resulting in NF-κB activation and; Mitogen-activated protein kinase (MAPK) cascade which culminates in the activation of the transcription factor Activator protein-1 (AP-1). IKKβ then phosphorylates the IκB which is ubiquitinated and degraded by the proteasome. NF-κB is translocated to the nucleus where it initiates transcription of pro-inflammatory cytokines and other genes involved in immune processes. NOD1/2 and TLR3 (via TIR-domain-containing adapter-inducing interferon-β (TRIF)) can also stimulate type-I interferon production via TANK-binding protein 1 (TBK) and interferon regulatory transcription factors 3/7 (IRF3/7).

NLR/TLR signaling to *Lm* infection primarily occurs in response to the detection of conserved microbe-associated molecular patterns (MAMPs) such as bacterial cell wall components. The NLRs NOD1 and NOD2 detect diaminopimelic acid (DAP) and muramyl dipeptide (MDP), respectively [29]; and TLR2, which forms heterodimers with TLR1, TLR6, or TLR10, and recognizes bacterial lipoproteins (LP), and lipoteichoic acids (LTA) [36,37]. TLR5, which detects bacterial flagella proteins, also responds to *Lm* derived flagellin [38]. However, because *Lm* suppresses flagella production at 37 °C, it is unclear whether TLR5 plays a significant role during infection [39]. In uninfected cells, in the absence of NLR/TLR activation, NF-κB is held in an inactive state by binding to its inhibitor IκB [28]. When NLRs/TLRs are stimulated, the signal is transmitted (via the adaptors Receptor-interacting serine/threonine kinase 2 (RIPK2) or Myeloid differentiation primary response 88/TIR-domain-containing adapter-inducing interferon-β (MyD88/TRIF) respectively) to the IκB kinase (IKK) complex composed of NF-κB essential modulator (NEMO), IKKα, and IKKβ. The activated IKK complex phosphorylates IκBα, which is ubiquitylated and degraded by the proteasome, freeing NF-κB to be translocated to the nucleus and perform its transcription factor function (Figure 1) [28,29]. NLR/TLR signaling also activates MAPK pathways via the TGFβ-activated kinase 1 (TAK1) which contributes to the induction of pro-inflammatory genes by downstream transcription factors such as Activator protein-1 (AP-1; Figure 1) [40,41].

In vivo studies in mice have demonstrated that NLR/TLR-mediated NF-κB responses are protective and important for restricting bacterial infection. However, because NLRs and TLRs are not ubiquitously or uniformly expressed across different cell types and tissues, the role of certain receptors in mediating inflammation can vary depending on the site of *Lm* infection. For instance, despite high expression of most NLRs and TLRs in phagocytic cells, the loss of one type of receptor in vivo cannot be functionally complemented by the presence of another [40,42,43,44,45]. During systemic *Lm* infection, NOD1 and TLR2 play a pronounced role in inflammatory responses, as mice which lack either receptor exhibit diminished inflammation, higher bacterial burden in the spleen and liver, and higher mortality [45,46,47,48]. On the contrary, during intragastric infection, NOD2 is suggested to have a dominant protective role [47,48]. However, the role of TLRs during intestinal infections in vivo is less clear as they are weakly expressed in the small intestine [49]. One study has shown that mice lacking the MyD88 adaptor poorly activate antimicrobial responses in the intestine, however, such a result could be explained by the role of this adaptor downstream of other receptors (i.e., IL-1 receptors (IL-1Rs)) [50].

**Figure 2:**
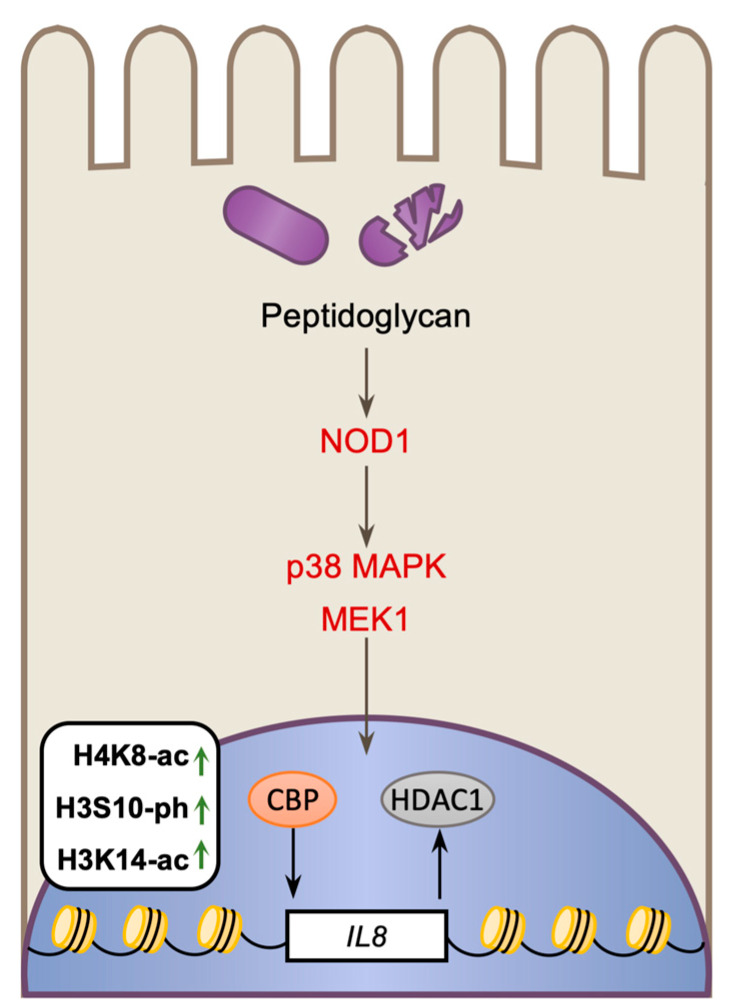
Infection-induced alterations to histone modifications in the host. *Lm* infection induces transcription of the *IL8* cytokine gene following the detection of cytoplasmic peptidoglycan by NOD1. Induction of *IL8* expression correlates with an increase in the histone modifications: H4K8-ac, H3S10-ph and H3K14-ac; which is dependent on p38 and MAPK/ERK kinase 1 (MEK1) signaling. The appearance of these histone modifications correlates with the recruitment of the acetyltransferase CREB-binding protein (CBP) and the loss of the histone deacetylase Histone deacetylase 1 (HDAC1) from the promoter of *IL8*.

Along with the initiation of broad transcriptional changes, receptor signaling also triggers histone modifications, which typically correlate with transcriptional activity, and promote pro-inflammatory responses [51,52]. In human umbilical vein endothelial cells (HUVECs), infection causes a specific increase in the levels of histone H4-Lys^8^ acetylation (H4K8-ac), histone H3 Ser^10^ phosphorylation, and Lys^14^ acetylation (H3K14-ac) at the promoters of pro-inflammatory genes, such as *IL8* [53]. These histone modifications are triggered by NOD1-induced MAP kinase signaling in response to cytosolic *Lm* (Figure 2) [54]; and temporally correlate with the recruitment of the acetyltransferase CREB-binding protein (CBP), and the loss of Histone deacetylase 1 (HDAC1), suggesting these enzymes may be responsible for regulating the histone acetylation. As IL8 is a key cytokine and chemoattractant for neutrophils, which are essential for the clearance of *Lm* in vivo, this process likely has a role in promoting a protective immune response [55]. Interestingly, histone modifications are only targeted to a subset of inflammatory genes, as the *IFNG* locus is not modified, despite being induced by infection. Therefore, the induction of histone modifications at specific genes likely fine tunes the host transcriptional response and imposes an extra layer of regulation.

### 2.2. Interferon-Regulated Transcriptional Profile

Interferon signaling is another major characteristic of *Lm*-induced transcriptional changes. Interferons (IFNs) play important roles in immunity and are grouped into type-I (IFN-β), type-II (IFN-γ), and type-III (IFN-λ) IFNs [56,57]. IFNs have primarily been studied for their antiviral role, however it is now appreciated that they also function in anti-bacterial responses; in fact IFN-β, γ, and λ have all been found to be released, and to activate ISGs in response to *Lm* infection [57]. Interestingly, some IFNs can produce variable immune outcomes depending on the context of infection, which in certain situations can enhance *Lm* infection [34].

**Figure 3:**
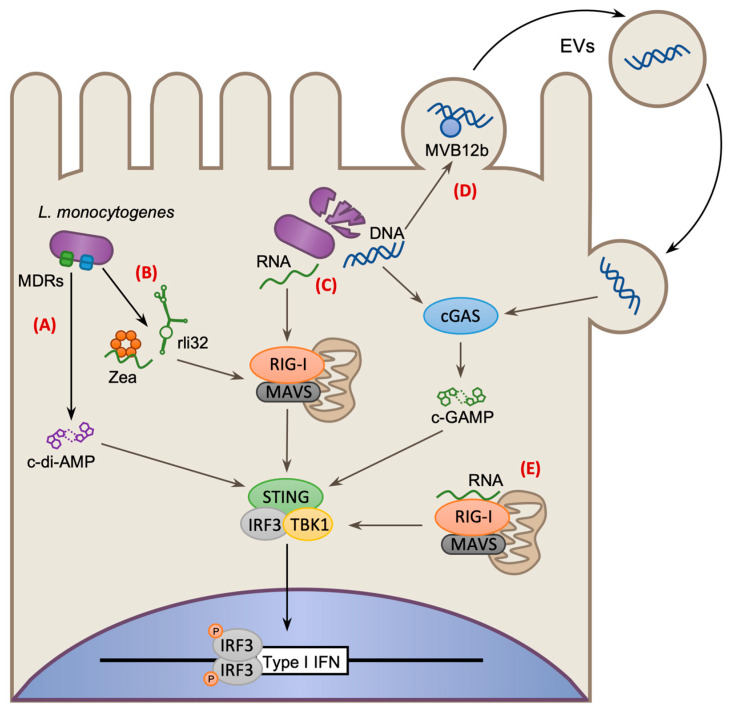
Mechanisms of type-I interferon (IFN) induction by *L. monocytogenes* via Stimulator of interferon genes (STING). Activated STING forms a platform for the recruitment of the interferon regulatory transcription factor 3 (IRF3) transcription factor and TANK-binding protein 1 (TBK1) kinase. IRF3 is phosphorylated by TBK1 and forms an active homodimer that translocates to the nucleus and initiates type-I IFN gene expression. (**A**) *Lm* secretes c-di-AMP through multidrug efflux pumps (MDRs). c-di-AMP is then recognized by STING, which activates and initiates type-I IFN expression via IRF3. (**B**) *Lm* secretes specific RNA molecules into the host to elicit type-I IFN. *Lm* can secrete sncRNAs such as rli32 and prophage-derived RNAs via a bacterial RNA-binding protein (RBP) called Zea. These result in activation of the cytosolic RNA receptor Retinoic acid-inducible protein I receptor (RIG-I), which can trigger type-I IFN expression via STING-dependent and independent mechanisms. (**C**) *Lm* releases DNA and RNA passively by bacterial autolysis or through active secretion. Cytoplasmic DNA is detected by the sensor cGAMP synthase (cGAS) which produces the cyclic nucleotide cGAMP to activate STING, whereas RNA is detected by RIG-I which signals to STING via Mitochondrial antiviral signaling protein (MAVS). (**D**) *Lm* DNA is packaged into host-derived extracellular vesicles (EVs) these can then fuse to neighboring cells to stimulate type-I IFN via cGAS and STING. (**E**) RIG-I can also signal independently of STING via TBK1 leading to the activation of IRF3.

In nonphagocytic cells, *Lm*-induced IFN-β signaling results in the activation of ISGs, such as the ubiquitin-like protein ISG15 which promotes cytokine secretion and restriction of bacterial intracellular growth [58]. Similar effects are also observed in vivo, for instance during gastrointestinal infection of mice, where IFN-β signaling helps mitigate the establishment of systemic infection by triggering rapid induction of protective cytokines [59,60]. Administering exogenous IFN-β can also elicit a protective host response during systemic infection, however this effect is limited to the first few hours of infection [61]. IFN-β is induced following direct sensing of bacteria-derived products. Both TLR2/3 and NOD2 have been shown to contribute to the induction of IFN-β in macrophages (Figure 1) [21,62]; however more notable are the roles of additional families of cytosolic PRRs (Figure 3) [20,22]. At the center of these responses is the host receptor Stimulator of interferon genes (STING), which induces IFN-β expression via its interactions with TANK-binding protein 1 (TBK1) and interferon regulatory transcription factor 3 (IRF3) [63]. Subsequently, IFN-β-induced activation of the Janus kinase/Signal transducers and activators of transcription (JAK–STAT) signaling pathway results in the induction of ISGs. STING acts as a central hub for multiple sensory pathways. In murine cells STING recognizes bacterial c-di-AMP which is secreted into the host via bacterial multidrug efflux pumps (MDRs), such as MdrM and MdrT (Figure 3A) [64,65]. By contrast, in human cells, STING is primarily activated in response to bacterial-derived cytosolic DNA and RNA, which are recognized by cGAMP synthase (cGAS) and Retinoic acid-inducible protein I receptor (RIG-I), respectively [66,67]. When cGAS binds DNA, it becomes activated and produces the cyclic nucleotide cGAMP, which subsequently activates STING (Figure 3C) [66]; whereas RIG-I forms a complex with Mitochondrial antiviral signaling protein (MAVS) and triggers IFN-β production through interactions with STING (Figure 3C) or via TBK1-induced IRF3 phosphorylation (Figure 3E) [67,68,69]. Despite the protective role of IFN-β in the gut [59,61], it has been shown to promote *Lm* pathogenesis during systemic infection by disrupting the generation of beneficial immune responses [34,60,70,71]. Interestingly, *Lm* possesses mechanisms that seemly function to actively elicit an IFN-β response, suggesting that, in some instances, its induction may represent a strategy used to enhance the susceptibility of the host to infection, a process discussed further on in this review (Section 3.1).

IFN-γ has been shown to be protective during many bacterial infections [72,73]. In the early stages of systemic *Lm* infection, cytokines IL-12 and IL-18 (secreted by infected macrophages) induce IFN-γ secretion from natural killer (NK)- and γδ T-cells [72,74,75,76]. IFN-γ-stimulated macrophages then induce ISGs, such as guanylate-binding proteins (GBPs), which promote direct killing of *Lm*, and initiate robust, protective antimicrobial responses [77,78,79]. In mice, IFN-λ expression is also observed in *Lm* infected placentas, suggesting it may have a role in regulating the fetoplacental barrier during infection [80].

## 3. Modification of Host Transcriptional Responses by *L. monocytogenes*

Induction of pro-inflammatory and antimicrobial genes are essential for the host to combat and clear bacterial infections. Therefore, it is unsurprising that bacterial pathogens have evolved diverse strategies to inhibit or subvert the expression of antimicrobial factors.

### 3.1. Exploitation of Antagonist Crosstalk in Interferon Signaling

In contrast to the response seen in the gastrointestinal tract, multiple studies have demonstrated that IFN-β can make the host more susceptible to systemic infection [34,70,71]. Notably, *Lm* possesses two mechanisms that can enhance its virulence by activating RIG-I-dependent IFN-β signaling [67,81,82]. In the first mechanism, *Lm* secretes small noncoding RNAs (sncRNAs) into the host cytosol, activating RIG-I, and resulting in IFN-β signaling which promotes *Lm* intracellular survival. One sncRNA, called rli32, displays particularly potent stimulation of IFN-β signaling, and its deletion greatly attenuates the growth and survival of *Lm* in macrophages (Figure 3B) [82]. The second mechanism requires the action of a bacterial RNA-binding protein (RBP) called Zea, which shuttles phage-derived RNA into the host cytosol to activate RIG-I, resulting in significant induction of IFN-β in epithelial cells (Figure 3B). However, during intravenous infection, Zea appears to reduce *Lm* virulence as *Δzea* strains display increased bacteria burden in the organs of infected mice, further illustrating that the temporal expression of IFN-β is tightly regulated in both pro- and anti-bacterial responses [81].

IFN-β is thought to sensitize the host to *Lm* infection by disrupting various immune processes. For instance, IFN-β has been shown to trigger T-cells apoptosis, resulting in a dampened immune response [83]; and suppress expression of CXC chemokines, causing reduced neutrophil infiltration and bacterial clearance [84]. This can also impact subsequent infections, as STING-dependent activation of IFN-β, during an initial *Lm* challenge, blocks the priming and expansion of CD8+ T-cells, rendering mice more sensitive to re-infection [85]. IFN-β also downregulates the expression the IFN-γ receptor in macrophages, thereby suppressing the protective properties of IFN-γ signaling [71,86]. This IFN type-specific antagonistic crosstalk is exploited by many bacterial pathogens in order to promote their infection [34,71,87,88].

Interestingly, *Lm* not only influences the transcription in infected cells, but also targets surrounding bystander cells. In infected macrophages, bacterial DNA was shown to be packaged and secreted in extracellular vehicles (EVs) by the host protein MVB12b, which is activated upon infection in a STING and TBK-1 dependent manner (Figure 3D). These EVs fuse with neighboring uninfected cells and stimulate cGAS-STING-dependent production of IFN-β, which promotes T-cell apoptosis in vivo [33]. However, although IFN-β can promote *Lm* pathogenesis, continuous and unrestricted induction of IFN-β can also negatively impact infection. Due to overexpression of the MDR MdrT, the *Lm* strain LO28 hyper-induces IFN-β production and, as a result, is attenuated during systemic infection [65]. This shows that the host-sensitizing effects of IFN-β has site and temporal specificity during infection, and must be tightly regulated by *Lm* in order to fine tune host responses [65]. Other bacteria such as *Mycobacterium leprae*, *Mycobacterium tuberculosis*, and *Salmonella enterica* Typhimurium have also been described to exploit the induction of IFN-β responses, implying this may be a common strategy employed during bacterial infection [34,87,88].

### 3.2. Virulence Factor-Induced Activation of Host Transcription

Host-mediated induction of classical pro-inflammatory responses usually occurs following the detection of MAMPs, which are often bacterial structural components or metabolites. However, a number of *Lm* virulence factors can also serve as activators of transcriptional processes in addition to their primary pathogenic function. As such, virulence factor-induced responses could either be an additional means of immune detection, which may serve to distinguish pathogenic from non-pathogenic organisms, or yet another host process that is exploited to suit the pathogen’s needs.

The pore forming toxin Listeriolysin (LLO), encoded by *hly*, primarily serves to promote bacterial escape from vacuoles; however it can also induce rapid NF-κB activation in endothelial cells, resulting in specific upregulation of adhesins and certain cytokines [89], which is also seen following treatment with purified recombinant LLO toxin. In contrast, non-hemolytic or Δ*hly* strains activate NF-κB to a slower and lesser degree, suggesting that early NF-κB responses in endothelial cells may, in large part, be dependent on LLO and not solely on MAMPs, as is the case in immune cells such as macrophages [90]. Further molecular studies have shown that LLO pores induce the activation of IKKβ, leading to NF-κB activation. Interestingly, this required Interleukin-1 receptor, type I (IL-1R1) but was independent of IL-1 cytokines, MyD88, and IL-1R-associated kinase (IRAK), which are essential in canonical IL-1R1 signaling [91]. Similarly, in Caco-2 intestinal epithelial cells, LLO-induced calcium influx triggers NF-κB activation resulting in the induction of IL-6. The same study also demonstrated that LLO activity preserves *IL-6* expression, resulting in persistent IL-6 secretion [92].

NF-κB activation in intestinal epithelial cells can also be influenced by Listeria adhesion protein (LAP), a multifunctional surface protein that promotes adhesion and translocation through the epithelium. LAP binds to surface expressed Hsp60, which activates pro-inflammatory NF-κB signaling and the Myosin light-chain kinase MLCK; thereby enhancing epithelium permeability, promoting bacterial intestinal translocation, and increasing systemic dissemination [93].

Internalin B (InlB), binds to its host receptor c-MET, and behaves as a functional mimic of the native agonist Hepatocyte growth factor (HGF). As well as mediating host cell invasion, InlB also stimulates multiple host signaling pathways, either while anchored to the bacterium, or as a free soluble cofactor [94,95]. Upon InlB binding, c-MET activates Phosphoinositide 3-kinase (PI3-K) which triggers AKT, JUN N-terminal kinase (JNK), and MAPK signaling cascades [94,95,96,97,98,99]. Many of these pathways communicate with host cell transcriptional machinery, thereby inducing a *Lm*-specific host program. In murine J774 macrophages, InlB has been shown to activate NF-κB via PI3-K and AKT, likely via IKKα [97,100,101]. Interestingly, the binding of InlB to c-MET occurs in a different region than HGF and generates more robust signaling cascade kinetics, raising the question whether the transcriptional responses induced by InlB and HGF are the same [98,99,102].

### 3.3. Direct Targeting of Host Transcription Factor Function

**Figure 4:**
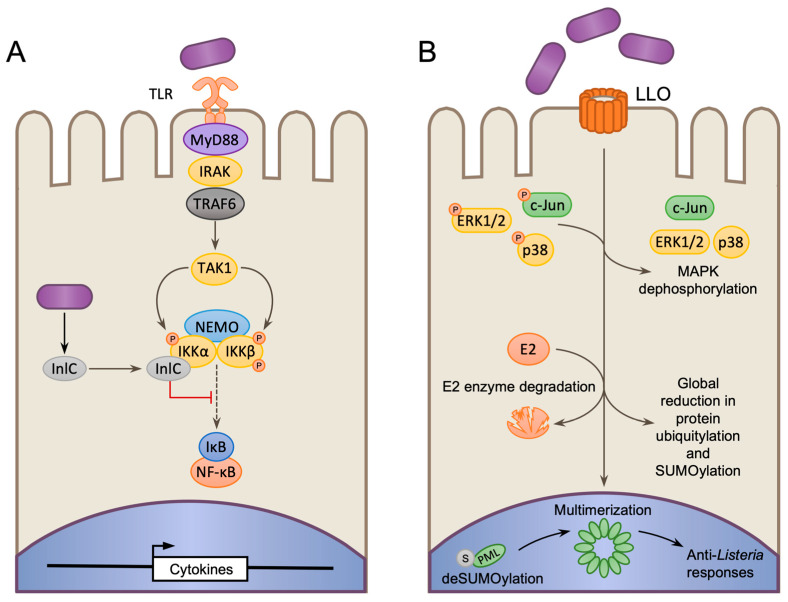
Altering host gene expression by disrupting transcriptional regulators. (**A**) The *Lm* effector internalin C (InlC) is produced and secreted when bacteria enter the host cytosol. InlC binds to IKKα and blocks IκB phosphorylation and degradation thereby inhibiting NF-κB nuclear translocation and the transcription of NF-κB-regulated gene. (**B**) Listeriolysin (LLO) blocks the phosphorylation of multiple enzymes in the Mitogen-activated protein kinase (MAPK) pathway thereby inhibiting the activation of downstream transcription factors such as c-Jun. LLO also induces proteolysis of ubiquitin and Small ubiquitin-like modifier (SUMO) E2-conjugating enzymes, causing a global decrease in ubiquitylated and SUMOlyated proteins, many of which, are transcription factors. The transcription regulator promyelocytic leukemia protein (PML) also undergoes deSUMOlyation in response to LLO, causing it multimerize and induce anti-*Listeria* responses.

Because NF-κB plays a central role in immune and inflammatory responses, it and its surrounding regulatory factors are common targets of bacterial virulence factors [103,104,105]. *Lm* is no exception and utilizes the virulence factor Internalin C (InlC) to disrupt NF-κB activation in macrophages [30]. InlC expression and secretion is induced upon the entry of *Lm* into the host cytosol, where it binds to the IKKα subunit of the IKK complex. This interaction suppresses phosphorylation and subsequent degradation of IκBα, thereby preventing the nuclear translocation of NF-κB, and blocking its transcriptional processes (Figure 4A). InlC therefore reduces the induction of pro-inflammatory cytokines during infection, and in mice, suppresses the recruitment of polymorphonuclear leukocytes to the site of infection [30]. In epithelial cells, IKKα has also been described to phosphorylate histone H3 to promote gene expression [106,107]. Therefore, InlC may also impact gene expression by affecting IKKα-induced histone phosphorylation, however further study is necessary. Recently, it has been shown that InlC is monoubiquitylated in host cells, which causes it to interact with the alarmin S100A9. Interestingly, this process promotes reactive oxygen species production by neutrophils and enhances host defense to infection, suggesting that InlC ubiquitylation might act as a host defense mechanism, that permits the induction of protective inflammatory signaling following the detection of bacterial virulence factors [108].

### 3.4. Indirect Targeting of Host Transcription Factor by Disruption of PTMs

PTMs such as phosphorylation, acetylation, ubiquitylation, and SUMOylation have the capacity to modify the activity of proteins, enabling rapid functional changes without the need for new transcription. As a result, disrupting PTM machineries is a common strategy used by pathogens to interfere with host processes [109]. For instance, LLO has been shown to trigger the dephosphorylation and inactivation of MAPK proteins p38, and Extracellular signal-regulated kinase 1/2 (ERK1/2) during *Lm* infection of trophoblast giant cells (Figure 4B). This inactivates c-Jun, a component of the AP-1 transcription factor, and promotes cell death through the downregulation of Heme oxygenase (HO)-1, a promoter of cell survival [110].

In non-phagocytic cells *Lm* also targets host ubiquitylation and SUMOylation. During infection, LLO triggers proteasomal-independent degradation of multiple ubiquitin E2 conjugating enzymes, and the sole Small ubiquitin-like modifier (SUMO) E2 enzyme UBC9, resulting in global loss of protein ubiquitylation and SUMOylation (Figure 4B) [111,112,113,114]. Ubiquitylation and SUMOylation regulate many proteins that control gene expression and inflammatory signaling [115,116]. Indeed, many of the proteins that are differentially modified during infection are transcription factors [111,113]. Therefore, by targeting these modifications, *Lm* could potentially have wide-reaching influence over multiple transcriptional and nuclear processes within the host, however, the full impact of targeting these proteins is yet to be determined [111]. The promyelocytic leukemia protein (PML), which regulates nuclear body formation, is one such target of LLO-induced deSUMOylation, and has been shown to play a role in restricting *Lm* growth. PML appears to act as a sensor of pore formation, as deSUMOylation causes PML multimerization, which in turn triggers the expression of antibacterial cytokines, and transcription factors that help the cell combat infection (Figure 4B) [117].

### 3.5. Modulation via Host Derived microRNAs

**Figure 5:**
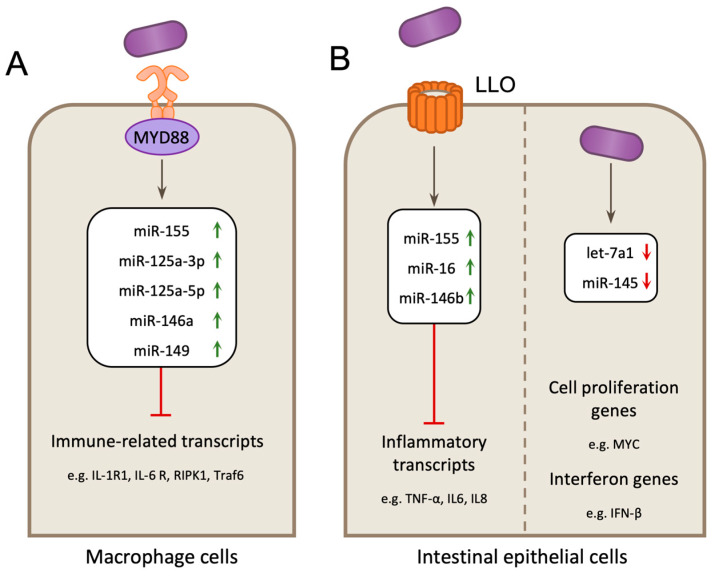
*L. monocytogenes* infection-induced changes in host microRNA (miRNA) expression. (**A**) In macrophage cells infection with *L. monocytogenes* causes significant upregulation of miRNAs miR-155, miR-146a, miR-125a-3p/5p and miR-149 in mouse bone marrow-derived macrophages. Upregulation was independent of Listeriolysin O (LLO) and required the host receptor adaptor MyD88 suggesting this occurs via MyD88-dependent receptor signaling (e.g., Toll-like receptors (TLRs) and Interleukin-1 receptor, type I (IL-1R)). These miRNAs target genes involved in the innate immune response. (**B**) In intestinal epithelial cells (Caco-2 cells) miR-155, miR-146b and miR-16 are induced upon infection, or treatment with purified LLO. miRNAs let-7a1 and miR-145 are down-regulated by infection in Caco-2 cells. let-7a1 is predicted to regulate expression of genes involved cell proliferation and metabolism such as Myc and miR-145 is predicted to target IFN-β.

MicroRNAs (miRNAs) are a class of small non-coding RNA, which post-transcriptionally repress gene expression by targeting complementary mRNAs for degradation by the RNA-induced silencing complex (RISC) [118]. Since their discovery, miRNAs have been established to regulate a number of physiological processes, including the regulation of specific host mRNAs during bacterial infection [118]. During *Lm* infection, many miRNAs are differentially expressed, however it remains to be shown whether these processes result in changes that are beneficial for *Lm* or the host. In macrophages, *Lm* induces the production of miR-155, miR-125a-3p, miR-125a-5p, miR-146a, and miR-149, all of which are implicated in regulating immune-related genes (Figure 5A). Upregulation of these miRNAs occurs independently of LLO but requires MyD88, suggesting that they are induced by MyD88-dependent receptors such as TLRs and IL-1Rs [119]. Similarly, *Lm* also alters miRNA expression in Caco-2 epithelial cells by inducing the expression of miR-155, and to a lesser extent miR-146b and miR-16 (Figure 5B). These up-regulated miRNAs are predicted to regulate multiple pro-inflammatory cytokines such as IL6 and IL8, and are also up-regulated in response to purified LLO toxin [120]. Two other miRNAs, let-7a1 and miR-145, are in fact suppressed during infection (Figure 5B). let-7a1 has been reported to regulate multiple transcripts of genes involved in the regulation of cell proliferation and metabolism, such as c-Myc [121], whereas miR-145 is predicted to regulate IFN-β [120]. By extrapolating their function, it can be hypothesized that infection-induced upregulation of these miRNAs could, in turn, suppress inflammatory responses by inducing the degradation of targeted cytokine mRNAs.

## 4. Targeting Host-Epigenetic Mechanisms

### 4.1. LLO-Induced Changes to Histone Modifications

**Figure 6:**
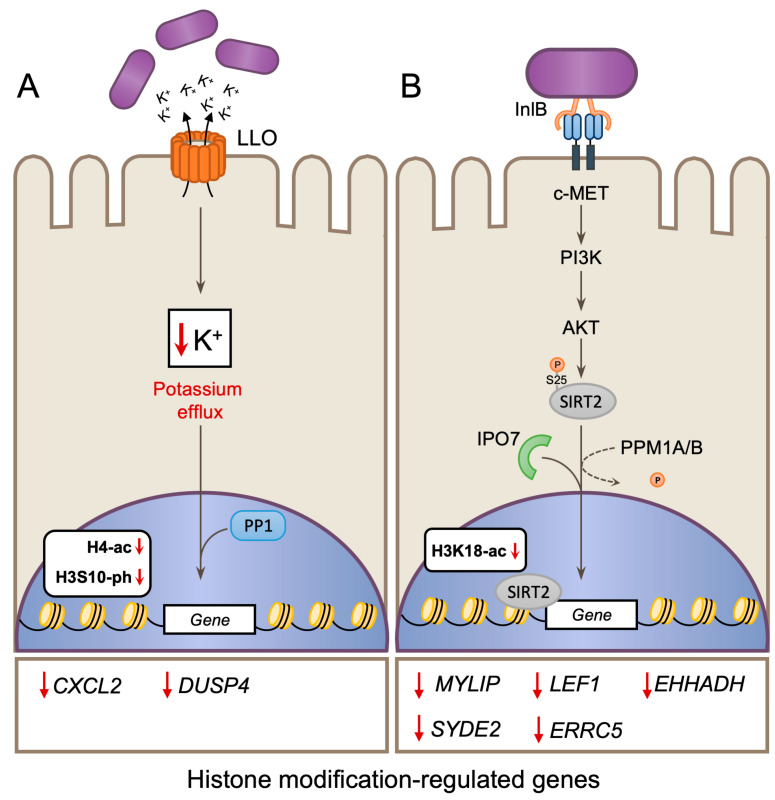
Infection-induced alterations to histone modifications in the host. (**A**) *Lm* infection or purified Listeriolysin O (LLO) induces global reduction in H4-ac and H3S10-ph which correlates with the down regulation of *CXCL2* and *DUSP4* genes. H3S10 dephosphorylation is dependent on LLO pore-induced potassium ion (K^+^) efflux [32] and requires the activity of the host Protein phosphatase 1 (PP1) [122]. (**B**) Internalin B (InlB) induced c-MET/Phosphoinositide 3-kinase (PI3K)/AKT signaling triggers redistribution of Sirtuin 2 (SIRT2) to host chromatin, deacetylation of H3K18 and gene repression. The host Importin 7 (IPO7) and SIRT2 dephosphorylation serine 25 (S25) by Protein phosphatases 1A/1B (PPM1A/PPM1B) are required for H3K18 deacetylation to occur. SIRT2 targets specific genes (e.g., MYLIP) which show concurrent enrichment of SIRT2, loss off H3K18 acetylation (H3K18-ac) and repressed gene expression.

LLO functions in many aspects of *Lm* infection and is often described as a “swiss army knife” [122]. It is unsurprising then that LLO can also alter host histone modifications and was in fact the first *Lm* virulence factor shown to do so [123]. In epithelial cells, infection or treatment with recombinant LLO toxin, triggers a global loss histone H4 acetylation (H4-ac) and histone H3S10 phosphorylation (H3S10-ph), both of which have been linked to active gene transcription (Figure 6A). In fact, LLO activity alters the expression of 146 host genes (47 repressed and 99 induced). Gene repression is correlated with reduced levels of H3S10-ph and H4-ac at gene promoters, while those that are induced have increased levels. Suppressed genes included the chemokine *CXCL2* and the phosphatase *DUSP4*, both of which have roles in the inflammatory response. Therefore, this mechanism may serve to dampen the immune response to *Lm* infection. Mechanistically, it has been determined that H3S10 dephosphorylation is dependent on LLO pore-induced potassium ion efflux [32] and can be triggered by other pore-forming toxins including perfringolysin O (PFO) and pneumolysin (PLY). Recently, both LLO and PLY induced dephosphorylation of H3S10-ph was shown to be dependent on the host Protein phosphatase 1 (PP1), which is itself activated by dephosphorylation in response to infection [124].

### 4.2. InlB-Dependent H3K18 Deacetylation by SIRT2

By virtue of its signaling capabilities via c-MET, the internalin InlB is able to trigger specific deacetylation of histone H3 at lysine 18 (H3K18) through hijacking of the NAD+ dependent deacetylase enzyme Sirtuin 2 (SIRT2). Activation of the c-MET/Phosphoinositide 3-kinase (PI3K)/AKT signaling axis during infection, causes SIRT2, which usually maintains a cytoplasmic localization, to be redistributed to host chromatin where it deacetylates H3K18 [125] (Figure 6B). This process is dependent on host Importin 7 (IPO7) [126]; and Protein phosphatases 1A/1B (PPM1A/PPM1B), which dephosphorylate SIRT2 at serine 25 to permit its association with chromatin. [127]. SIRT2 activity during infection causes widespread transcriptional reprogramming of the host, resulting in the repression of 272 genes [125]. Examining repressed genes by chromatin immunoprecipitation (CHIP) PCR, showed that SIRT2 becomes enriched at gene promoters and correlates with H3K18 deacetylation. Conversely, genes whose expression remains unaltered by infection showed no change in SIRT2 or H3K18-ac, suggesting that SIRT2-dependent H3 deacetylation is gene loci specific. Deacetylation of H3K18 appears to play an important role during infection, as inhibition or knockdown of either SIRT2 or PPM1A/PPM1B suppresses bacterial intracellular survival without affecting bacterial invasion [125,127]. Similarly, SIRT2 deficiency also restricts *Lm* survival during infection in vivo. Certain SIRT2-repressed genes, such as *LEF1* and *CXCL12*, have roles in regulating immune responses. Therefore, the modulation of SIRT2 by *Lm* may serve as a general strategy to block or down-regulate the induction of immune and antimicrobial responses at both cell-intrinsic and systemic levels.

### 4.3. Chromatin-Targeting Bacterial Factors

**Figure 7:**
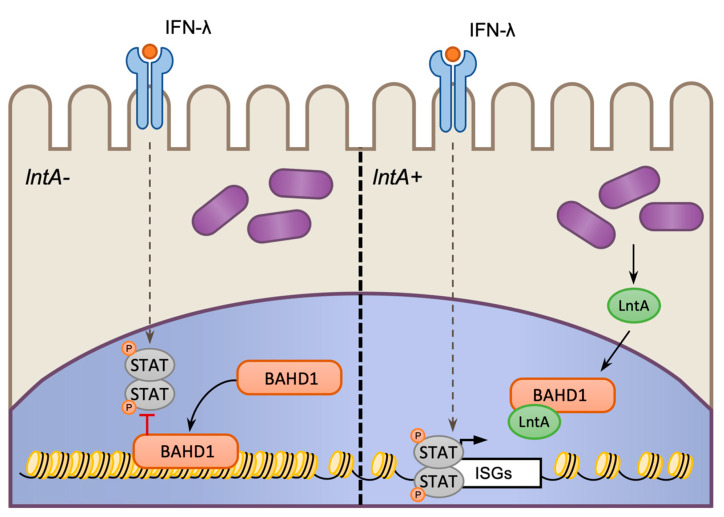
Regulation of host gene expression via LntA-targeting of host BAHD1 protein. *Lm* infection induces the production of IFN-λ which stimulates the expression of IFN-stimulated genes (ISGs). During infection with *Lm* which lack the nucleomodulin LntA the repressor complex protein BAHD1 is activated, binds to ISGs and blocks their expression by Signal transducers and activators of transcription (STAT) factors (**left**). When LntA is expressed it is secreted into the cytosol of infected cells and binds to BAHD1, preventing it from interacting with chromatin and blocking ISG expression (**right**).

Virulence factors such as LLO and InlB enable *Lm* to alter the transcriptional output of cells by subverting or hijacking native host signaling processes. However, *Lm* also produces factors that directly target the nucleus and chromatin structure. *Lm* has specialized effectors termed nucleomodulins, which specifically enter the host cell nucleus and target nuclear/chromatin factors that regulate gene expression. The first such factor to be described in *Lm* was LntA, a secreted protein that modulates the activity of the host heterochromatinization factor BAHD1 (Figure 7). In vertebrates, BAHD1 interacts with transcription factors, such as SP1, at CpG-rich promotors, where it acts as a platform for the recruitment of chromatin silencing factors such as heterochromatin protein 1 (HP1α, β, and γ), MBD1, KMT1E, HDAC5, and CHD1. Once recruited, these factors cause heterochromatin formation and gene silencing of cell proliferation and survival genes as well as ISGs during *Lm* infection [128,129,130].

Although LntA is regulated by PrfA and SigB, the timing and the location of its expression in vivo remains poorly understood [129,131]. When LntA is expressed, it localizes to the nucleus and binds to BAHD1, preventing it from repressing the expression of ISGs, and resulting in IFN-λ-dependent gene expression. However, when LntA is not expressed, BAHDI is able to bind to promoters and block transcription of ISGs downstream of IFN-λ signaling, which is maintained during infection (Figure 7). Interestingly, deletion or constitutive expression of LntA reduces bacterial virulence resulting in lower bacterial burdens in infected mice. Thus, LntA appears to have an important role in fine-tuning IFN-λ-dependent transcriptional responses at specific points during the infection process [129,130].

## 5. Conclusions and Perspectives

During infection, mammalian cells undergo significant transcriptional reprogramming to bring about the necessary inflammatory and antimicrobial responses to combat the pathogen. These processes require the concerted action of receptors, signaling pathways, transcriptional factors, and epigenetic mechanisms to engage a robust and protective transcriptional response. Concurrently, bacterial pathogens utilize diverse mechanisms to manipulate host transcriptional pathways at all levels, in order to create a more favorable niche for survival and replication. These dynamics are well represented during *Lm* infection, which as discussed above, triggers widespread global transcriptional changes in its host. In the broadest sense, transcriptional responses either act in favor of the host (triggered by conserved bacterial MAMPS), or the pathogen (triggered by specialized bacterial virulence factors).

However, in some cases this distinction is not always clear, highlighting an interesting dichotomy in host–pathogen interactions whereby the line between host- and pathogen-regulated responses can be hard to distinguish. Despite not being considered virulence factors, *Lm* utilizes both passive and active mechanisms to release molecules such ci-di-AMP or rli32, which are not directly involved in specific pathogenic function but nonetheless impact infection. Likewise, the fact that virulence factors, such as LLO, also induce typically anti-microbial responses raises the same questions. On one hand, such events could be considered as the “detection” of a virulence factor, resulting in activation of an antimicrobial response; whereas on the other, it could function to promote bacterial virulence. For instance, in the case of InlB, AKT-mediated activation of NF-κB has also been implicated in blocking apoptosis, therefore this mechanism may serve to help promote a pro-survival response in infected cells, thereby promoting the intracellular cycle of the pathogen [101]. Similarly, in non-phagocytic cells, InlB is implicated in activating pro-survival signaling via the ERK1/2 kinases and STAT3 transcription factor. Although a precise transcriptional network of InlB signaling is yet to be determined, treating endothelial cells with recombinant InlB has been demonstrated to block the induction of apoptosis in response to angiotensin II, suggesting that these pathways could function to promote host cell survival during infection, thereby maintaining the intracellular niche of *Lm* [132]. In these instances, whether a transcriptional response is deliberately activated as a means of host manipulation can be difficult distinction to make, and is further complicated by the fact that blocking virulence factor activity often perturbs infection and pathogenesis (i.e., invasion or phagosome escape). Another level of complexity is introduced when the presence of other bacterial species, such as the components of the microbiota, are considered. Recent advances in microbiome research have shown that the gut microbiota can influence intestinal homeostasis and immunity by modulating host epitranscriptomic [133] and epigenetic modifications [134,135,136]. *Lm* is known to produce bacteriocins that kill other bacteria to promote its own infection, therefore, it may also be able to affect the transcriptional profile of its host by targeting specific members of the host gut microbiome [137,138]. Though this requires further study, it could represent yet another mechanism through which *Lm* influences the transcriptional profile of its host.

Studying the dynamic interface of transcriptional regulation during infection allows us to discover how pathogens exploit their host to promote their own pathogenesis, and sheds light on potential new targets for therapeutics, in both the pathogen and the host. Though still in its early stages of development, the concept of host-directed immunomodulatory therapies is an attractive alternative to bacterial targeted treatments such as antibiotics, as resistance to such treatment is, theoretically, more difficult to develop. The study of epigenetics brings further scope and potential to the development of such treatments, whereby they could be designed to be long lasting and cross protective for multiple pathogens [139,140].

In summary, despite the significant progress made in the study of transcriptional responses to infection, current microarray studies do not appropriately illustrate the more nuanced changes that can be regulated by *Lm* virulence factors, and many questions still remain. For instance, how heterogenous are bacterial-induced changes within infected tissue? It is possible that these changes function through precise fine-tuning of specific genes, allowing their effects to be global without having a drastic impact on the transcriptional profile of the host. This could be particularly true of the epigenetic-regulated changes, as these appear to display significant gene loci specificity. For example, this is illustrated by LLO-mediated suppression of CXCL2, which is also induced as part of the NF-κB transcriptional profile [21]. Furthermore, it remains to be demonstrated how transcriptional changes induced by pathogen virulence factors mechanistically confer an advantage to the pathogen within the host; and in the context of epigenetic mechanisms, how long they may persist, and whether they act on cell intrinsic, local or systemic levels. From these examples, it is clear that the innate immune response to bacterial infection is greatly influenced by transcriptional and epigenetic changes, however these can be highly varied and contextually nuanced. Studying these heterogenous responses will help better our understanding of human pathology during infectious disease.
